# Survival and Mortality of Pumas (*Puma concolor*) in a Fragmented, Urbanizing Landscape

**DOI:** 10.1371/journal.pone.0131490

**Published:** 2015-07-15

**Authors:** T. Winston Vickers, Jessica N. Sanchez, Christine K. Johnson, Scott A. Morrison, Randy Botta, Trish Smith, Brian S. Cohen, Patrick R. Huber, Holly B. Ernest, Walter M. Boyce

**Affiliations:** 1 Karen C. Drayer Wildlife Health Center, School of Veterinary Medicine, University of California Davis, Davis, California, United States of America; 2 The Nature Conservancy, San Francisco, California, United States of America; 3 California Department of Fish and Wildlife, Valley Center, California, United States of America; 4 Information Center for the Environment, University of California Davis, Davis, California, United States of America; U.S. Geological Survey, UNITED STATES

## Abstract

Wide-ranging large carnivores pose myriad challenges for conservation, especially in highly fragmented landscapes. Over a 13-year period, we combined monitoring of radio collared pumas (*Puma concolor*) with complementary multi-generational genetic analyses to inform puma conservation in southern California, USA. Our goals were to generate survivorship estimates, determine causes of mortality, identify barriers to movement, and determine the genetic and demographic challenges to puma persistence among >20,000,000 people and extensive urban, suburban, and exurban development. Despite protection from hunting, annual survival for radio collared pumas was surprisingly low (55.8%), and humans caused the majority of puma deaths. The most common sources of mortality were vehicle collisions (28% of deaths), and mortalities resulting from depredation permits issued after pumas killed domestic animals (17% of deaths). Other human-caused mortalities included illegal shootings, public safety removals, and human-caused wildfire. An interstate highway (I-15) bisecting this study area, and associated development, have created a nearly impermeable barrier to puma movements, resulting in severe genetic restriction and demographic isolation of the small puma population (n ~ 17–27 adults) in the Santa Ana Mountains west of I-15. Highways that bisect habitat or divide remaining “conserved” habitat, and associated ongoing development, threaten to further subdivide this already fragmented puma population and increase threats to survival. This study highlights the importance of combining demographic and genetic analyses, and illustrates that in the absence of effective measures to reduce mortality and enhance safe movement across highways, translocation of pumas, such as was done with the endangered Florida panther (*P*. *c*. *coryi*), may ultimately be necessary to prevent further genetic decline and ensure persistence of the Santa Ana Mountains population.

## Introduction

Many large carnivores have been extirpated from substantial portions of their historic range, and extant populations are threatened by habitat loss and fragmentation, and conflict with humans [1]. Human population growth expected over the next century exacerbates these threats [2–5], and exurban development will have substantial impacts on habitat that today is still relatively intact [6]. Because large carnivores pose myriad challenges for conservation in urbanizing landscapes, we began a long-term study of pumas (*Puma concolor*) in 2001 in southern California, USA, to provide quantitative insights and guidance for conservation of pumas and other large carnivores in human-dominated habitats.

Pumas, also known as mountain lions, cougars, or panthers, are wide-ranging carnivores that historically occurred throughout the Americas. Humans have extirpated or greatly reduced puma numbers in much of their former range in the past 200 years [2]. The only documented breeding population of pumas remaining in the eastern United States is in Florida, where a small population of federally endangered pumas (Florida panthers–*Puma concolor coryi*) persists, largely because their endangered status spurred intensive management including translocation and genetic introgression [7, 8]. In the western United States, pumas are hunted for sport in several states, but there is considerable controversy and uncertainty about the long-term consequences of hunting on population persistence. For example, a recent study of heavily-hunted and semi-protected puma populations in Utah [9] did not detect a compensatory decrease in natural mortality in response to heavy hunting pressure, and concluded that uncertainties in the functional relationship between natural and anthropogenic mortality could lead to biased conclusions and mismanagement.

In California, pumas are considered a “specially protected mammal” and hunting is prohibited [10]. Despite these protections, recent genetic studies of pumas in southern California show that the genetic viability and long-term persistence of some populations are in jeopardy [11, 12]. The threats facing pumas in southern California—habitat loss, increased conflict with humans, demographic isolation, and genetic restriction [11–15]—mirror the challenges facing large carnivores in urbanizing landscapes around the world [3], and long-term studies intended to guide conservation and management in these settings are difficult and expensive to conduct. We addressed this information gap by conducting a 13-year study of pumas in the California south coast ecoregion, USA, a biodiversity hotspot [16, 17] with a growing population of >20,000,000 people [18].

Sandwiched between the sprawling metropolitan areas of greater Los Angeles and San Diego, much of the available puma habitat in our study area is not protected from new highways and development, and is subject to ongoing habitat loss and fragmentation [15]. Our goals for this study were to generate survivorship estimates and cause-specific mortality data for pumas in this region, and identify options for improving survivorship and facilitating movement within and among conserved and non-conserved areas. This demographic study builds upon and complements our recent genetic analysis [11], and provides the essential ecological context for understanding the causes and potential solutions to the genetic restriction we found in pumas in the Santa Ana Mountains.

## Materials and Methods

### Statement

We operated under Protocol 10950/PHS, Animal Welfare Assurance number A3433-01, with capture and sampling procedures approved in Protocol number 17233 by the Animal Care and Use Committee at the University of California, Davis, and Memoranda of Understanding and Scientific Collecting Permits from the California Department of Fish and Wildlife (CDFW). Permits and permissions for access to conserved lands where captures and monitoring were conducted were obtained from CDFW, California Department of Parks and Recreation, The Nature Conservancy, United States (U.S.) Fish and Wildlife Service, U.S. Forest Service, U.S. Bureau of Land Management, U.S. Navy / Marine Corps, U. S. Fish and Wildlife Service, Orange County Parks Department, San Diego County Parks Department, Riverside County Parks Department, San Diego State University, University of California—Riverside, Audubon Starr Ranch, Vista Irrigation District, Rancho Mission Viejo / San Juan Company, Sweetwater Authority, California Department of Transportation, the City of San Diego Water Department and Parks Department, and the Irvine Ranch Conservancy. Anesthetic drug combinations used in capture procedures were either teletamine / zolazapam (Telazol) or medetomidine / ketamine at dosages prescribed in the scientific literature.

### Study Area and Population

**Fig 1:**
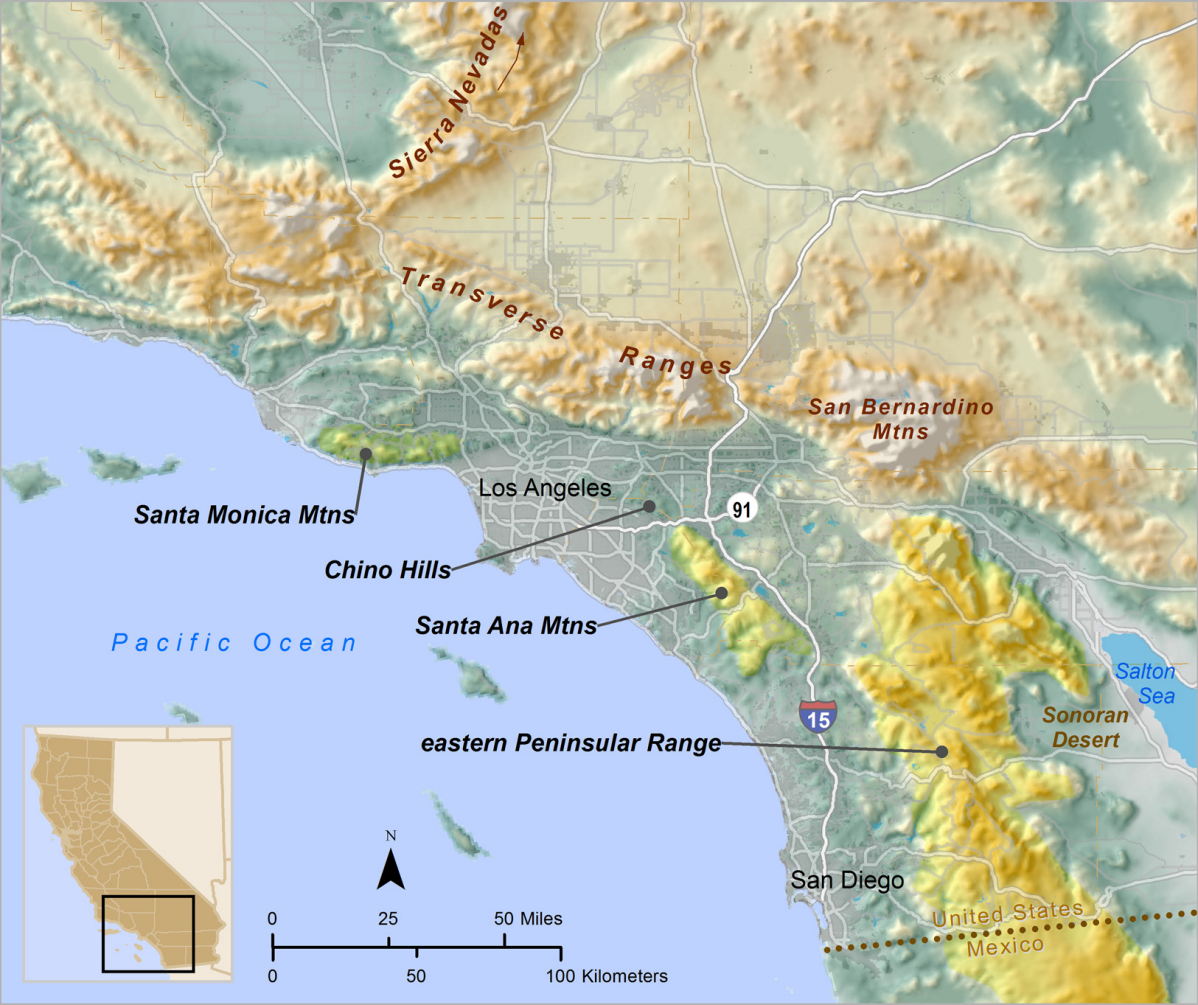
Puma study area in southern California, USA, and regional context. The focal area of this study includes the Santa Ana Mountains and the eastern Peninsular Range. Inset shows location within California.

The study area encompassed the Santa Ana Mountains (a portion of the Peninsular Ranges) and the remainder of the Peninsular Mountain Ranges and surrounding foothills to the east (hereafter referred to as the eastern Peninsular Range). These areas constitute the majority of occupied puma habitat in southern California south of greater Los Angeles (Fig 1). Pumas are the primary large carnivore remaining in the study area since grizzly bears (*Ursus arctos californicus)* were extirpated in the early 1900s [19, 20].

An extensive and growing network of roads, some carrying more than 250,000 vehicles per day, encircles and fragments the study area [21] (Fig 1). Interstate highway 15 (I-15) connects the greater Los Angeles, Riverside, and San Diego metropolitan areas, and the highway and associated development have been hypothesized to be a barrier to puma movement between the Santa Ana Mountains in the west and the eastern Peninsular Ranges [11, 13, 22, 23]. Therefore, we assigned pumas that were captured or found dead west of I-15 to a putative “Santa Ana Mountains” source population, and those east of I-15 to a putative “eastern Peninsular Range” source population.

Land use varies considerably across the study area, with the eastern Peninsular Ranges generally having less intensive development and more rural, undeveloped, and protected lands. Burdett et al. [15] classified land use and urbanization in the study area into five categories: protected public lands (55% of the study area), private undeveloped (9.5%), rural (14.4%; >16.18 ha per housing unit), exurban (15.7%; 0.68–16.18 ha per housing unit), and suburban/urban (5.4%; <0.68 ha per housing unit). The Santa Ana Mountains have substantial protected public lands, but new highway construction, development, and land use practices tend to be much more intensive immediately adjacent to remaining high quality puma habitat [15].

### Capture and Monitoring Methods

We captured, marked, and monitored radio collared pumas from 2001 through 2013. Pumas were captured primarily using baited cage traps [24], and to a lesser extent using hounds or foothold snares [25, 26]. Each captured animal was tattooed in one ear with a unique numerical identifier (“marked”) and a numbered tag was placed in the opposite ear. Age was determined from dental characteristics and body morphometrics as described in Ashman et al. [27] and Laundre et al. [28, 29]. We classified pumas <18 months as kittens, 18–30 months as subadults, and >30 months as adults [30]. We applied Very High Frequency (“VHF”; MOD500 Telonics, Mesa, AZ), and/or Global Positioning System (“GPS”; Simplex P-1D, Televilt, Lindesberg, Sweden; TGW 3580, Telonics, Mesa, AZ; GPS4400S, GPS3300S, and GlobalstarTrack S, Lotek, Ontario, Canada) radio collars to pumas if their body weight exceeded 22.7 kg. GPS locations were collected at varying time intervals from every 5 minutes to every 6 hours depending on specific study objectives.

### Puma Movements

We hypothesized that the Pacific Ocean, Sonoran Desert, major highways, and urban centers would form barriers that constrained pumas in our study area into one or more discrete populations. To test this hypothesis, we examined movements of radio collared pumas from 2001–2013 to determine the degree of interchange within and between the Santa Ana Mountains and eastern Peninsular Range populations, and whether monitored pumas emigrated out of the entire study area. We were particularly interested in determining if ongoing habitat fragmentation had created or hardened existing barriers, and in evaluating puma movements relative to corridors or linkages identified through previous modeling efforts. These included: 1) the east-west “Santa Ana—Palomar Mountains Linkage” across I-15 that connects the Santa Ana Mountains and eastern Peninsular Range puma populations [31]; 2) the “Coal Canyon Corridor” under California State Route 91 (SR-91) linking the Santa Ana Mountains and the Chino Hills to the north [17, 32]; and 3) the “Parque-to-Park Linkage” connecting California and Baja California, Mexico to the south [33].

### Mortality and Survival Analyses

The distribution of radio collared pumas in the populations west and east of the I-15 freeway were compared using the Fisher exact test to determine if they differed by sex or age class, and a two-sample t-test was used to determine if they differed by average age at entry or exit to the study (in months), or the average number of days monitored (STATA IC 13.0, STATACorp, College Station, Texas, USA). A P value of ≤ 0.05 was used as the cutoff for significance for all analyses.

Deaths of radio collared pumas from 2001–2013 were detected when VHF or GPS data indicated a lack of movement, and the cause of death was determined by field investigation and necropsy. In addition, we combined mortality data for our marked (n = 36) pumas with “unmarked” (n = 218) pumas that were confirmed to have died in the study area from 1981–2013 based on CDFW records. Age determination was less precise for unmarked animals due to conditions of carcasses at discovery or variable experience of reporting parties in the aging of pumas. Therefore, we classified unmarked pumas as either subadults (≤30 months) or adults (>30 months) [30].

We compared long-term trends in the number of pumas being killed under depredation permits in our dataset to the total number of puma mortalities across all of California during the same time period, as reported by CDFW [34], by plotting 5-year simple moving averages for each dataset. For each calendar year, the number of puma mortalities was averaged for the current year and previous 4 years. This allowed us to smooth short-term fluctuations and visualize long-term trends in the data.

Radio collared pumas entered the study on the date they were first captured, and exited on the date of mortality or the last date of detection by radiotelemetry. The number of days each animal was monitored was calculated as the time between entry and exit dates. Cause of death was determined at necropsy for both marked and unmarked animals unless the state of carcass decomposition precluded definitive diagnosis. In those cases, cause of death was classified as “unknown”. Because numbers of at-risk individuals varied across the course of the study, a formal cause-specific mortality analysis was not possible [35]. However, source population, sex, and age class (at time of mortality) were evaluated in both marked and unmarked populations for associations with the various causes of mortality using the Fisher exact test (STATA IC 13.0, STATACorp, College Station, Texas, USA).

We estimated survival using the known-fate model in Program MARK (Version 7.1) [36]. We chose a set of 8 models a priori for analysis that included combinations of population, sex, and age class (at the time of collaring) as parameters. Support for each model was assessed using Akaike’s Information Criterion (AIC), corrected for small sample size (AIC_c_). The *sin* link function was used to run all models. If no model was clearly superior to all others (AICc weight >90% and**Δ**AIC >2) [36–38], we performed model averaging to reduce the uncertainty in our parameter estimates.

We used a Cox proportional hazards model (STATA IC 13.0) to evaluate the relationship of biologically important covariates (source population, sex, age, and year of mortality) to the length of time pumas survived during the study. Adult age class (>30 months old) and mortality years 2007 and 2008 were used as reference categories in the models. Staggered entry into the study was addressed by including the Andersen-Gill formulation [39, 40]. The Breslow approximation method was used to address tied failure times [41], and Schoenfeld residuals were used to test the proportional hazards assumption that relative risk for each variable of interest was the same for the duration of the study.

## Results

### Puma Monitoring and Movements

Our analyses included 74 pumas that were captured, marked, and radio collared between March 2001 and December 2013 (Table 1). The distribution of these marked pumas in the eastern Peninsular Range (n = 43) and Santa Ana Mountains (n = 31) did not differ significantly by sex, age class, average age at entry or exit to the study, or the average number of days monitored.

**Table 1:** Demographic characteristics of radio collared pumas in southern California, USA from 2001–2013.

Variable	Number of pumas		
	Total	Santa Ana Mountains	Eastern Peninsular Ranges
**Sex**			
Females	37	17	20
Males	37	14	23
**Outcome**			
Survived	38	18	20
Died	36	13	23
**Age class at entry**			
<18 months	19	6	13
18 to 30 months	19	9	10
>30 months	36	16	20
**Age class at exit**			
<18 months	2	0	2
18 to 30 months	16	7	9
>30 months	56	24	32

**Fig 2:**
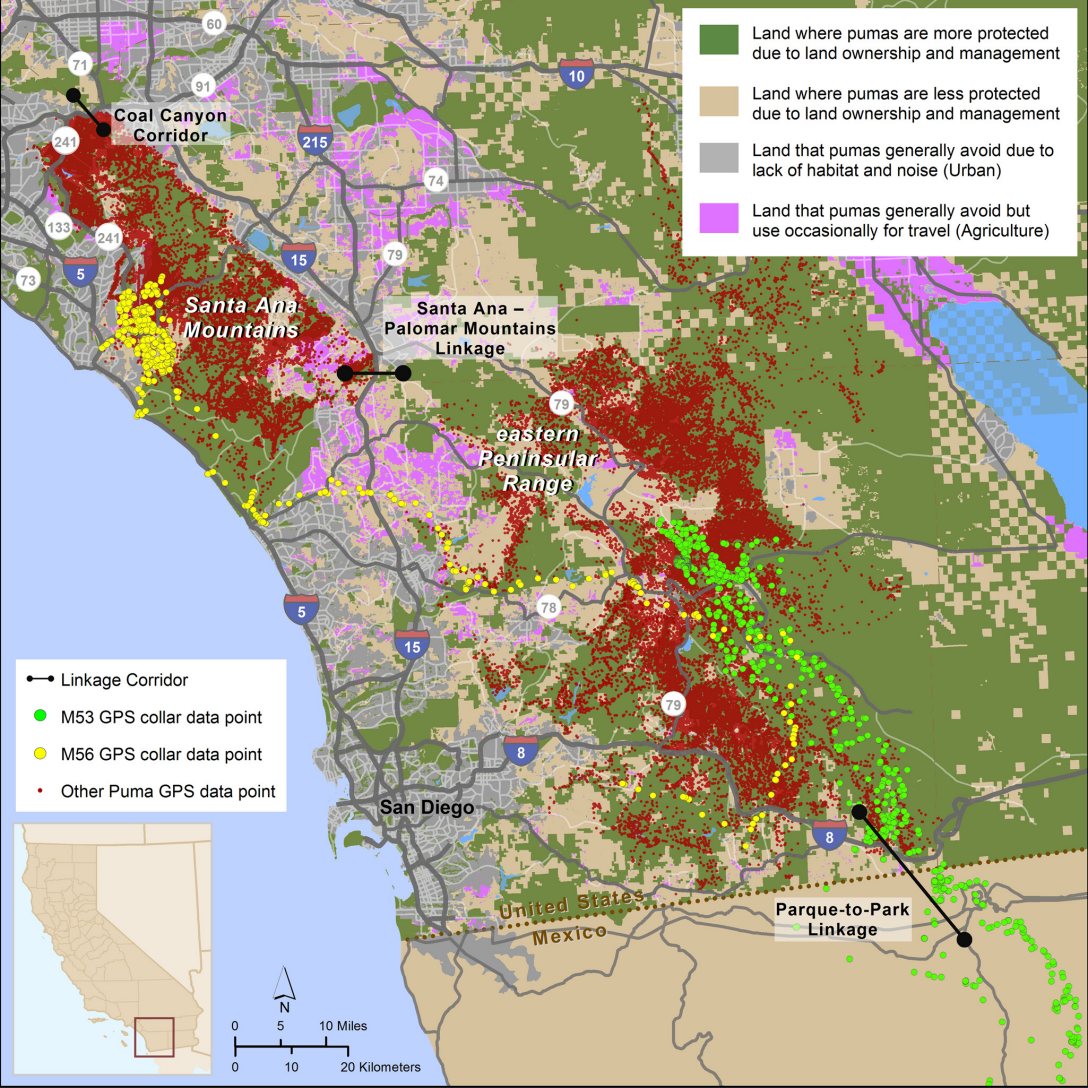
Puma GPS collar data points collected from 2001–2013 in southern California, USA. GPS collar data points are overlaid on lands classified (by color) based on their relative levels of puma protection and typical usage. Primary linkages within the study area are noted and GPS collar data points from pumas M53 and M56 are highlighted. Inset shows location within California.

We detected numerous long distance (>80 km) movements by radio collared pumas from 2001–2013, but only one radio collared puma moved out of the overall study area. In 2009, a young adult male (M53) traveled approximately 150 km south from his capture site in the eastern Peninsular Range, utilizing the Parque-to-Park Linkage to cross the U.S.-Mexico border (Fig 2). He reached a point 70 km south of the border before returning to his original location in the U.S. Several other radio collared pumas were detected near, but not across, the U.S.-Mexico border (Fig 2).

Radio collared puma movements between the eastern Peninsular Range and Santa Ana Mountains were limited, indicating the pumas in these regions formed relatively discrete populations. In 2010, a dispersing sub-adult male (M56) crossed I-15 from west to east several miles south of the proposed Santa Ana Mountains—Palomar Mountains Linkage (Fig 2), but he was killed 25 days later for depredating domestic sheep.

None of the radio collared pumas used the Coal Canyon undercrossing beneath SR-91 at the northern tip of the Santa Ana Mountains to move into the Chino Hills to the north (Fig 2). However, two unmarked pumas were killed from 2001–2013 while attempting to cross this major freeway within 3 km of the Coal Canyon undercrossing.

### Puma Mortality

**Fig 3:**
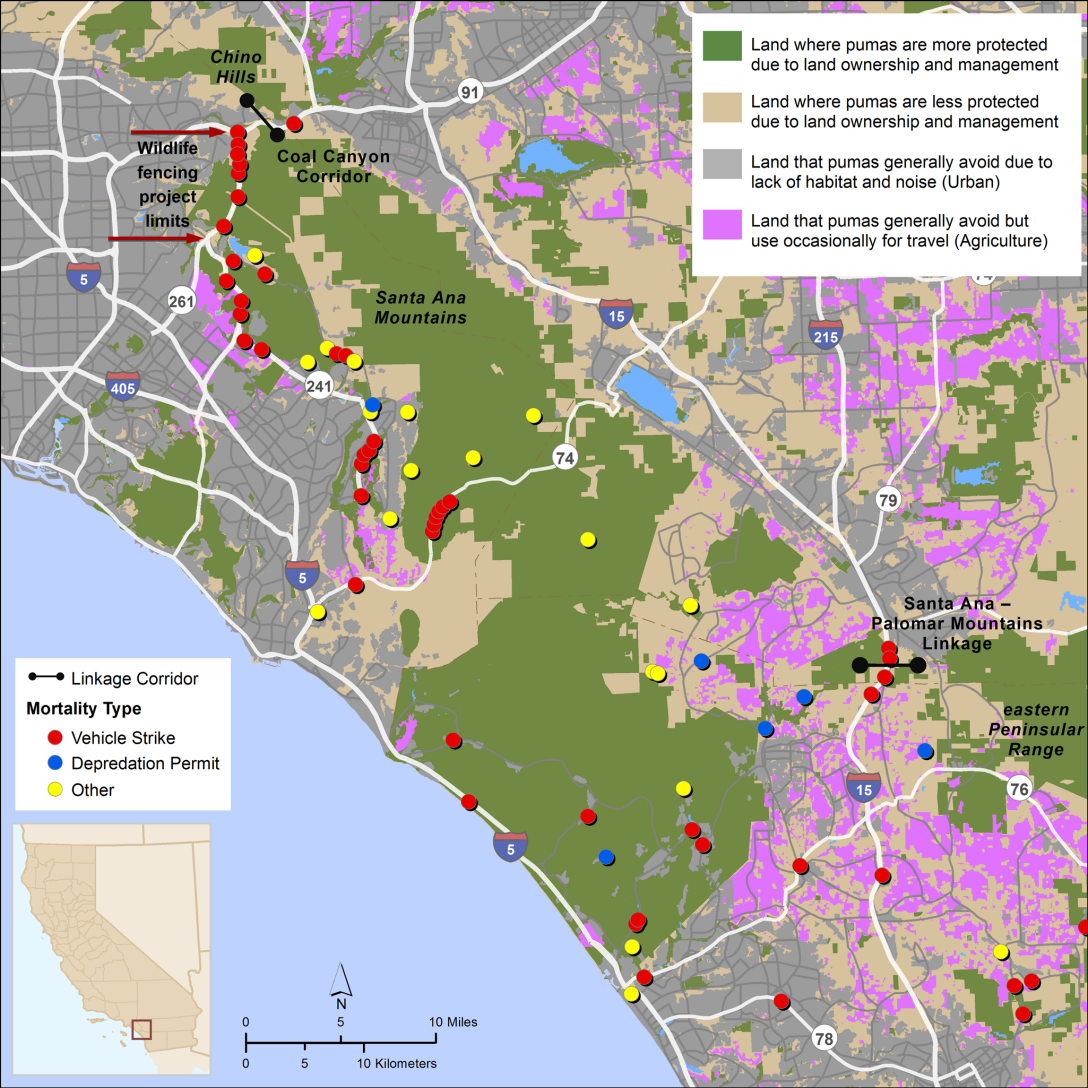
Sites and causes of puma mortalities in the Santa Ana Mountains, 1981–2013. The Coal Canyon Corridor and Santa Ana—Palomar Mountains Linkage are noted, as well as the limits of a wildlife fencing project on SR 241. Inset shows location within California.

**Fig 4:**
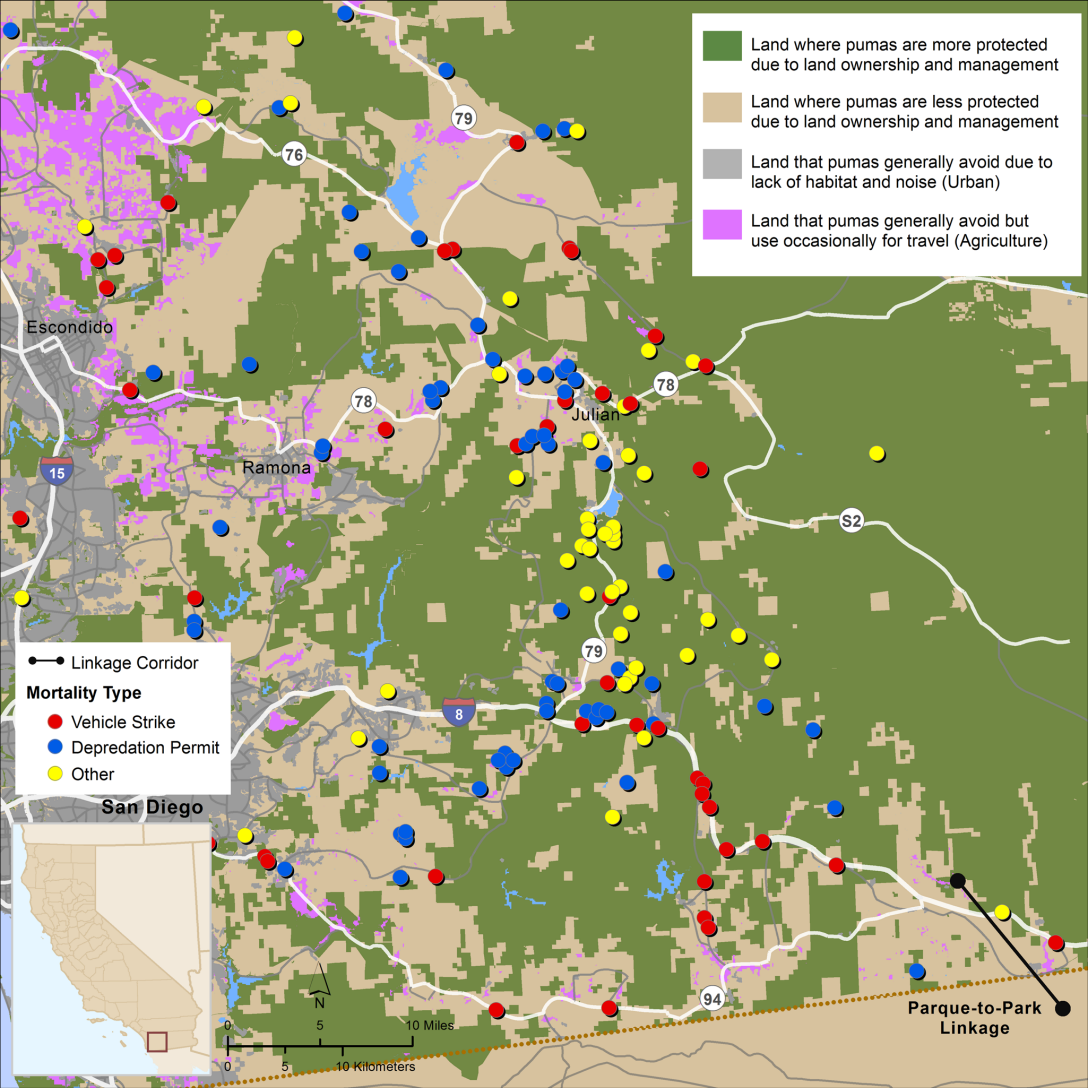
Sites and causes of puma mortalities in the eastern Peninsular Range, 1981–2013. Area depicted is generally east of Interstate 15, with the Parque to Park linkage noted. Inset shows location within California.

The number of pumas alive (n = 38) vs. dead (n = 36) by the end of the study did not differ between source populations, sexes, or age class at entrance or exit (Table 1). Though time-at-risk and sample size constraints prevented a formal cause-specific mortality analysis, we report here proportional mortality of both marked and unmarked pumas over the entire study period. Over the course of the entire study period, proportional mortality due to vehicle collisions and depredation permits was greater than for all other causes of mortality. Other known sources of mortality included disease, illegal shooting, arson-caused wildfire, public safety removal, and intraspecific aggression (Table 2). Proportional mortality due to vehicle strikes and depredation permits differed between pumas from the eastern Peninsular Range and Santa Ana Mountains populations (*P* = 0.034), but did not differ by sex or age class. In fact, all mortalities of marked pumas due to depredation permits occurred in the eastern Peninsular Range, while 60% of mortalities due to vehicle collisions were in the Santa Ana Mountains (Table 2; Figs 3 and 4). With the exception of vehicle strikes and depredation, the data were too sparse to evaluate relationships between specific causes of mortality and various risk factors.

**Table 2:** Proportions and numbers of radio collared pumas that died from different causes in Southern California study areas from 2001–2013.

Cause of mortality	Total	Santa Ana Mountains	Eastern Peninsular Ranges
Vehicle Strike^a^	0.28 (10)	0.46 (6)	0.17 (4)
Depredation Permit^a^	0.17 (6)	0.00 (0)	0.26 (6)
Killed illegally	0.11 (4)	0.23 (3)	0.04 (1)
Disease suspected	0.11 (4)	0.08 (1)	0.13 (3)
Disease confirmed	0.06 (2)	0.00 (0)	0.09 (2)
Fire	0.06 (2)	0.08 (1)	0.04 (1)
Public safety	0.03 (1)	0.00 (0)	0.04 (1)
Killed by other puma	0.03 (1)	0.00 (0)	0.04 (1)
Capture related	0.03 (1)	0.00 (0)	0.04 (1)
Unknown	0.14 (5)	0.15 (2)	0.13 (3)
Total	36	13	23

^a^ Mortalities secondary to depredation permits and vehicle strikes

differed between the two populations (P = 0.034; Fisher’s exact test).

**Fig 5:**
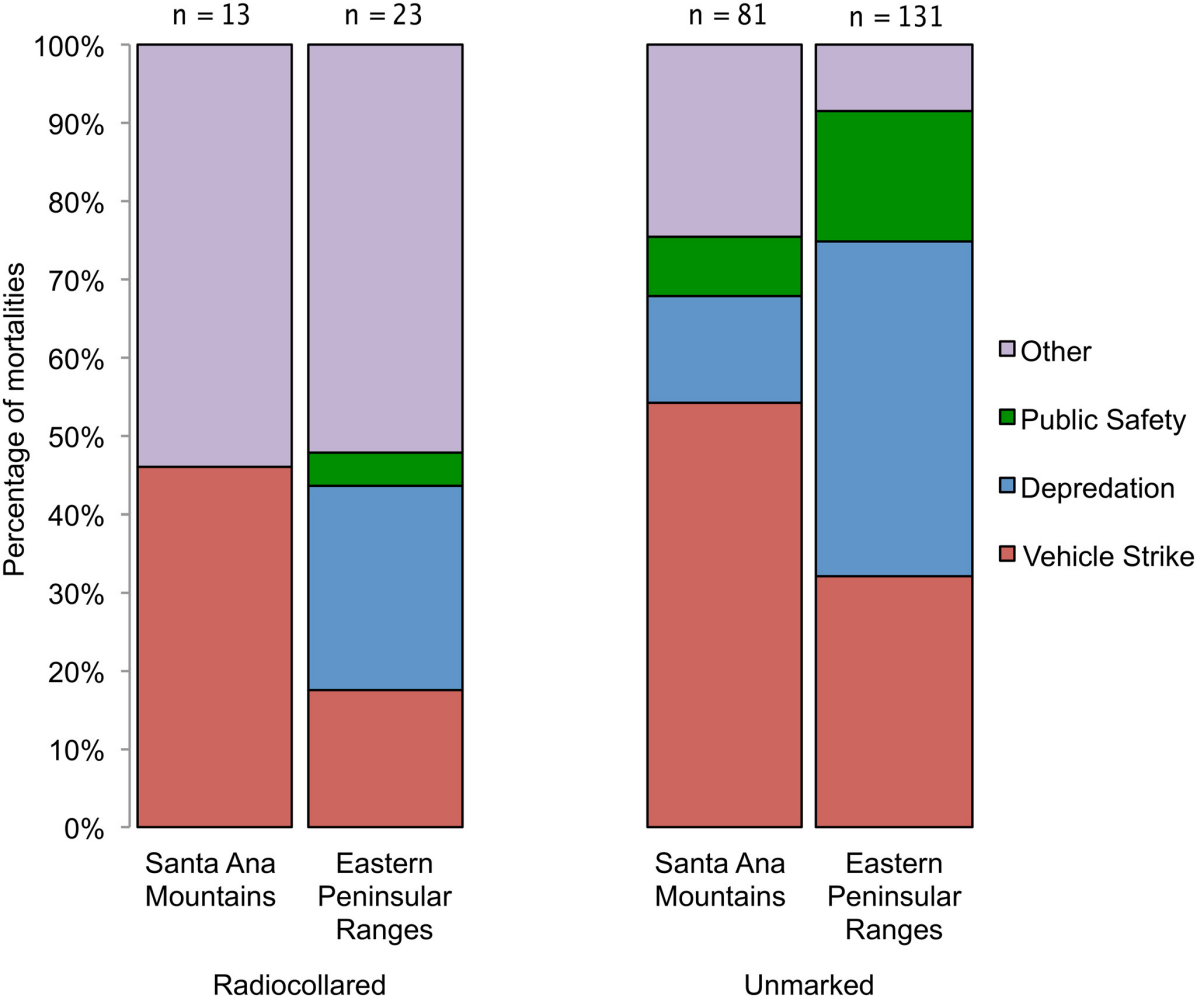
Percentages of pumas dying from different causes of mortality in southern California, USA. Mortalities of radiocollared (n = 36; 2001–2013) and unmarked (n = 212; 1981–2013) pumas were assigned to the Santa Ana Mountains or the eastern Peninsular Ranges subpopulation. Six unmarked pumas were not included in this figure because they were found on the I-15 freeway and could not be assigned to a population.

**Fig 6:**
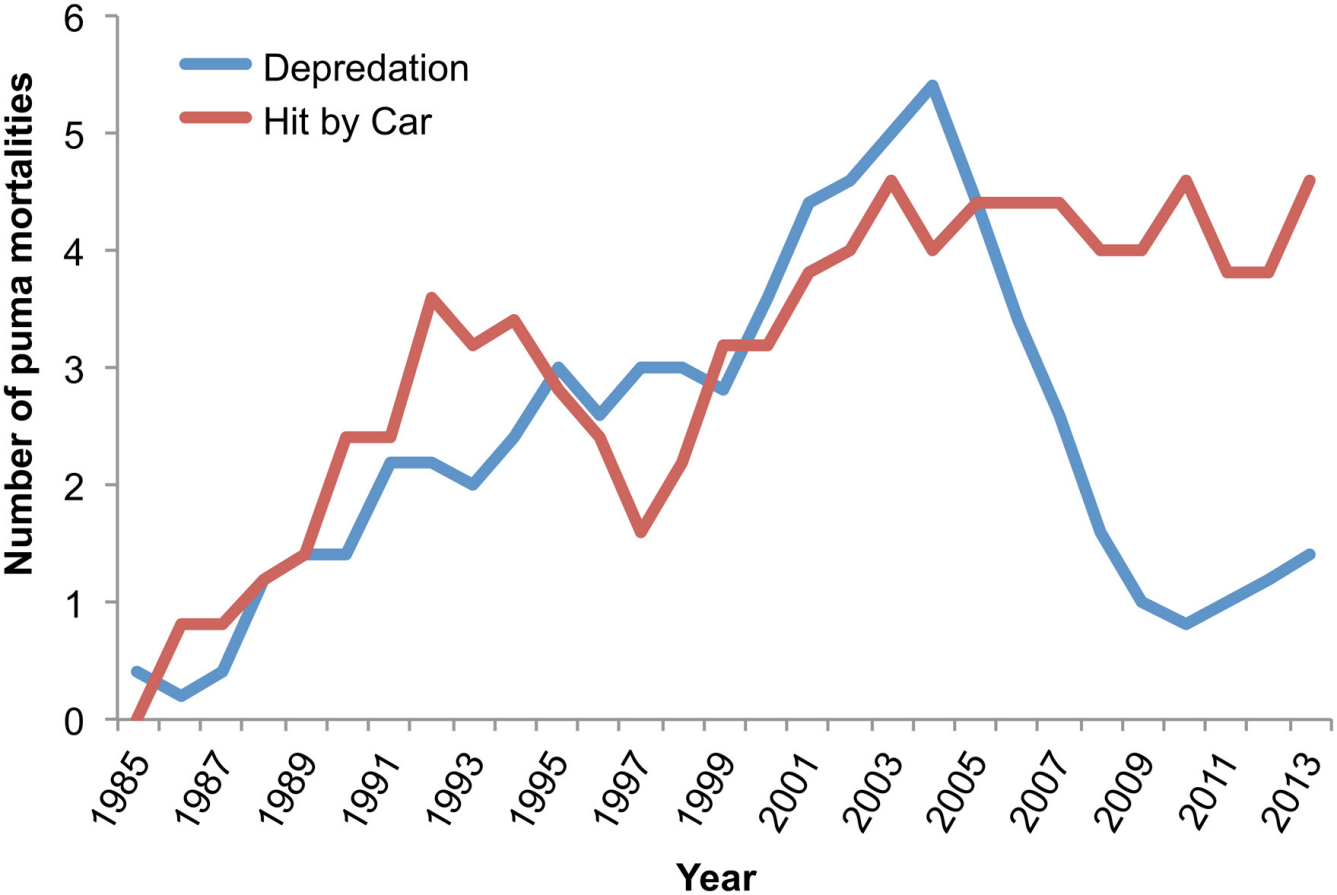
Pumas killed secondary to vehicle collisions or depredation permits from 1981–2013 in southern California, USA. Five year moving average of pumas killed secondary to vehicle collisions or depredation permits (n = 174) in the Santa Ana Mountains and eastern Peninsular Ranges.

In the combined dataset (marked and unmarked pumas; n = 254), proportional mortality due to depredation permits was approximately 3.4 times higher for males than females (54M:16F; *P =* 0.001), while proportional mortality due to vehicle collisions was equal for both sexes (45M:45F). In the combined dataset, proportional mortality varied between the eastern Peninsular Range and Santa Ana Mountains populations (*P* < 0.001). There were more mortalities due to depredation permits in the eastern Peninsular Range (n = 62, compared to n = 11 in the Santa Ana Mountains), and almost equal numbers of mortalities due to vehicle collisions in the two populations (n = 46 in the eastern Peninsular Range, compared to n = 50 in the Santa Ana Mountains; Fig 5). Mortality varied substantially year by year in the combined dataset, but the trend for mortalities due to depredation permits tended to increase from 1981 to 2004 and then began to decline, before trending upward again through 2013 (Fig 6), a pattern generally similar to that seen in CDFW’s graph of statewide depredation data (http://www.dfg.ca.gov/wildlife/lion/depredation.html). In contrast, the trend for deaths due to vehicle collisions increased steadily through 2013, with no decline or downward trend detected after 2004 (Fig 6). Vehicle mortalities occurred throughout the study area (Figs 3 and 4), however the majority of vehicle-related mortalities in the Santa Ana Mountains occurred on California State Highway 241 (SR-241) and SR-74, two highways that traverse puma habitat, and I-15 in the Santa Ana Mountains to Palomar Mountains Linkage area (Fig 3).

### Puma Survival

Survival estimates were calculated using data from the 74 radio collared pumas that were monitored from 2001–2013 for a total of 29,578 puma days (mean = 400, SE = 38 days per animal). Using the known-fate model within Program MARK, the estimated mean annual survival rate was 55.8%, (95% CI = 44.5–65.6%). In the most parsimonious model survival was constant across populations, sexes, and ages (“S(.)”; Table 3). Two models had ΔAIC values <2: model “S(Sex)” which included sex as a parameter (model likelihood = 0.43), and model “S(Population)” which included population as a parameter (model likelihood = 0.37; Table 3). These models yielded annual survival estimates of 58.6% for females and 52.5% for males across the entire study area, and 56.5% for the Santa Ana Mountains population and 55.4% for the eastern Peninsular Range population across all sexes and age groups (Table 3). Due to the distribution of AICc weights among the top models (Table 3), we performed model averaging of similarly parameterized models but did not detect any differences among groups (95% CI of survival estimates overlapped).

**Table 3:** Results of the known-fate model (Program MARK) for survival (S) for radio collared pumas in southern California, USA.

Model	AICc	ΔAICc	AICc Weights	Model Likelihood	Number Parameters	Deviance
S(.)	397.21	0.00	0.4668	1.00	1	327.25
S(Sex)	398.89	1.68	0.2012	0.43	2	326.93
S(Population)	399.20	1.99	0.1724	0.37	2	327.24
S(Age)	400.24	3.03	0.1026	0.22	3	326.27
S(Population*Sex)	402.69	5.48	0.0301	0.06	4	326.72
S(Population*Age)	403.68	6.48	0.0183	0.04	6	323.70
S(Sex*Age)	405.22	8.01	0.0085	0.02	6	325.24
S(Population*Sex*Age)	414.57	17.37	0.0001	0.00	12	322.52

Survival rates varied widely among years, and the Cox proportional hazards model identified calendar years 2001, 2003 2005, 2006, and 2009 as having significantly higher hazard ratios compared to years 2007 and 2008 (Table 4). All other covariates, including source population, sex, and age at entry were not significantly associated with time to death. Evaluation of the proportional hazards assumption for the final model based on a test of Schoenfeld residuals indicated that the relative risk for each variable of interest, after including year as a variable in the model, did not differ for the duration of the study (*P =* 0.99).

**Table 4:** Variables related to time to death in the Cox proportional hazards model of survival of radio collared pumas in southern California, USA.

Covariate^a^	Hazard Ratio	SE	Z	P>|z|	95% CI
**Age at Entry** ^b^					
<18 months	0.53	0.28	-1.21	0.23	0.19–1.48
18–30 months	1.56	0.75	0.93	0.35	0.61–3.99
**Year at Exit** ^c^					
2001*	149.20	209.78	3.56	0.00	9.48–2347.27
2002	5.16	6.85	1.24	0.22	0.38–69.63
2003*	9.86	8.34	2.71	0.01	1.88–51.73
2004	5.41	6.98	1.31	0.19	0.43–67.91
2005*	12.75	11.51	2.82	0.01	2.18–74.76
2006*	7.10	6.82	2.04	0.04	1.08–46.64
2009*	8.02	7.79	2.14	0.03	1.20–53.77
2010	1.70	1.71	0.53	0.60	0.24–12.16
2011	3.03	4.03	0.84	0.40	0.23–40.89
2012	6.16	6.14	1.82	0.07	0.87–43.44
2013	3.55	3.48	1.29	0.20	0.52–24.23

*Covariates significant at P ≤ 0.05.

^a^The final model controlled for population and sex which were not significantly associated with time to mortality

^b^Oldest age class (>30 months old) designated as reference category.

^c^Years 2007 and 2008 designated as reference category

## Discussion

This 13-year study demonstrates the high risk of mortality for pumas associated with fragmentation and urbanization, and coupled with our genetic analyses [12], we conclude that puma persistence in this human-dominated landscape is threatened [42–46]. Annual puma survival rates for radio collared pumas in the Santa Ana Mountains (56.5%) and eastern Peninsular Range (55.4%) were very low from 2001–2013, and were similar to those in heavily hunted populations [2, 9, 43]. Indeed, annual survival rates for our study population were lower than rates for pumas in the peri-urban Santa Monica Mountains population northwest of Los Angeles (>75%) [12], and are within the range that is considered a threat to persistence of puma populations [13].

Our movement data (this paper) and our genetic findings [11] support the hypothesis that pumas in the Santa Ana Mountains and eastern Peninsular Range effectively form two subpopulations, bisected by an interstate highway and neighboring development. Our data demonstrate that both subpopulations had low survival; and though proportional mortality is a crude measure of causes of death in a population, the major causes of puma proportional mortality differed between these areas. Depredation permits were the most common proportional mortality factor in the eastern Peninsular Range and primarily affected males, whereas vehicle strikes were the main source of proportional mortality in the Santa Ana Mountains, affecting males and females equally. Conservation biologists have long expressed concern about demographic and genetic isolation of pumas in the Santa Ana Mountains [11, 13], and this study, coupled with our companion genetic study [11], provides a comprehensive view of the fractured demographic and genetic connectivity among pumas in this region.

Ernest et al. [11] concluded that Santa Ana Mountains pumas monitored in this study “had high average pairwise relatedness, high individual internal relatedness, a low estimated effective population size, and strong evidence of a bottleneck and isolation from other populations in California.” Genetic restriction and isolation were pronounced even though limited gene flow did occur from the eastern Peninsular Ranges into the Santa Ana Mountains. Genetic analysis showed that a male puma (M86) captured in the Santa Ana Mountains was likely born in the eastern Peninsular Ranges, and successfully migrated into the Santa Ana Mountains during our study. This male, and two females (F61 and F89) captured in the Santa Ana Mountains, were the likely parents of four pumas born in the Santa Ana Mountains in 2010–2011 (M91, F92, M93, and M97) [11]. However, this is the only evidence of successful genetic interchange between the two populations during the study period other than an 8 month old kitten (F102) [11] killed by a car in the Santa Ana Mountains in August 2003. In 13 years, none of the pumas radio collared in the eastern Peninsular Range were observed to move west into the Santa Ana Mountains, and the single radio collared male that did move from the Santa Ana Mountains into the eastern Peninsular Range was killed for depredating domestic sheep within weeks of crossing I-15. This suggests that the estimated 17–27 adult pumas in the Santa Ana Mountains [13] have become an insular population, much like the small population of pumas located in the Santa Monica Mountains [12]. The combination of small population size, limited potential for immigration of new individuals (male and female) into the area, female mortality rates that are similar to males, and negative effects of genetic restriction [11], collectively put the Santa Ana Mountains population at risk for demographic collapse [13, 22, 47].

Southern California has been the focus of multiple regional-scale conservation planning efforts aimed at protecting a network of natural habitats, among extensive urban, suburban, and exurban development and a burgeoning population of >20,000,000 people [48]. Pumas have been a focal species for these efforts because of their ecological value, their inherent value to humans, and their utility as surrogates for other wide-ranging taxa in conservation planning [13, 22, 42, 49–52]. Despite some concerted conservation efforts (e.g. [14]), this study shows that pumas are currently subject to high levels of human-caused mortality, and that wildlife corridors that facilitate safe movement through the landscape are lacking or insufficient. These threats will only grow worse without further action. For example, additional urban development is underway or proposed on both the east and west sides of I-15 in the Santa Ana–Palomar Mountains Linkage (Figs 2 and 3) [53, 54], and 14,000 new homes and associated highways will be constructed at the south end of SR-241 in the center of puma habitat in the Santa Ana Mountains [55].

Conserving core habitat areas and functional wildlife corridors has been the main focus of conservation efforts for pumas in southern California [15, 31, 32, 56, 57] and coordinated regional action in the form of targeted investment in habitat protection is especially urgent to maintain viability of the Santa Ana Mountains population. However, our analysis highlights that land protection alone will not be sufficient to ensure puma persistence in the region. Also important will be directed focus on improving road infrastructure to facilitate safe wildlife crossings, and reducing depredation conflicts that precipitate puma deaths. Options for enhancing movements across I-15 and other highways include protection of additional lands on both sides of the highway, improving or adding large culverts, adding exclusionary fencing [58] such as that currently being constructed on SR 241 (Fig 3) [59], and possibly constructing vegetated overpasses for wildlife use [60, 61]. Strategies to reduce mortalities stemming from depredation permits include education activities to promote wider use of predator-proof enclosures for their domestic animals during the crepuscular periods and at night [62–65]. A focus on land protection, roadway and wildlife crossing design, and landowner outreach will be critical for persistence of puma in southern California, and may well be a formula for conserving large carnivores in highly populated and fragmented landscapes generally.

The combination of long term field monitoring of radio collared animals coupled with genetic analyses was critical for understanding puma biology and providing directions for conservation efforts in southern California. The movement of puma M86 across I-15 from the eastern Peninsular Ranges may aid in the genetic rescue of the Santa Ana population, but only if his offspring survive and reproduce. To date, only one of his four known offspring are still alive in the wild—a female with two dispersal-age offspring, and evidence points towards pumas being less likely to successfully navigate this human-dominated landscape in the future. In the absence of effective measures to reduce mortality and enhance safe movement across highways, translocation of pumas, such as was done with the Florida panther [66], may ultimately be necessary to prevent further genetic decline and assure persistence of the Santa Ana Mountains population.
